# Association of Liver Enzymes and Computed Tomography Markers of Liver Steatosis with Familial Longevity

**DOI:** 10.1371/journal.pone.0091085

**Published:** 2014-03-14

**Authors:** Michiel Sala, Lucia J. M. Kroft, Boudewijn Röell, Jeroen van der Grond, P. Eline Slagboom, Simon P. Mooijaart, Albert de Roos, Diana van Heemst

**Affiliations:** 1 Department of Radiology, Leiden University Medical Center, Leiden, the Netherlands; 2 Department of Gerontology and Geriatrics, Leiden University Medical Center, Leiden, the Netherlands; 3 Department of Molecular Epidemiology, Leiden University Medical Center, Leiden, the Netherlands; 4 Netherlands Consortium for Healthy Ageing, Leiden, the Netherlands; University College London, United Kingdom

## Abstract

**Objective:**

Familial longevity is marked by enhanced peripheral but not hepatic insulin sensitivity. The liver has a critical role in the pathogenesis of hepatic insulin resistance. Therefore we hypothesized that the extent of liver steatosis would be similar between offspring of long-lived siblings and control subjects. To test our hypothesis, we investigated the extent of liver steatosis in non-diabetic offspring of long-lived siblings and age-matched controls by measuring liver enzymes in plasma and liver fat by computed tomography (CT).

**Research Design and Methods:**

We measured nonfasting alanine transaminase (ALT), aspartate aminotransferase (AST), and Υ-glutamyl transferase (GGT) in 1625 subjects (736 men, mean age 59.1 years) from the Leiden Longevity Study, comprising offspring of long-lived siblings and partners thereof. In a random subgroup, fasting serum samples (n = 230) were evaluated and CT was performed (n = 268) for assessment of liver-spleen (L/S) ratio and the prevalence of moderate-to-severe non-alcoholic fatty liver disease (NAFLD). Linear mixed model analysis was performed adjusting for age, gender, body mass index, smoking, use of alcohol and hepatotoxic medication, and correlation of sibling relationship.

**Results:**

Offspring of long-lived siblings had higher nonfasting ALT levels as compared to control subjects (24.3 mmol/L versus 23.2 mmol/L, p = 0.03), while AST and GGT levels were similar between the two groups. All fasting liver enzyme levels were similar between the two groups. CT L/S ratio and prevalence of moderate-to-severe NAFLD was similar between groups (1.12 vs 1.14, p = 0.25 and 8% versus 8%, p = 0.91, respectively).

**Conclusions:**

Except for nonfasting levels of ALT, which were slightly higher in the offspring of long-lived siblings compared to controls, no differences were found between groups in the extent of liver steatosis, as assessed with liver biochemical tests and CT. Thus, our data indicate that the extent of liver steatosis is similar between offspring of long-lived siblings and control subjects.

## Introduction

Non-alcoholic fatty liver disease (NAFLD) is the most common chronic liver disease in Western countries and is associated with metabolic risk factors such as obesity, diabetes mellitus, and dyslipedimia [1]. NAFLD is prevalent in more than one-third of the elderly [2], while prevalence may increase up to 69% in type 2 diabetes patients [3].

Hepatocyte dysfunction due to liver fat accumulation may interfere with insulin action and cause hepatic insulin resistance [4]. Accordingly, the liver enzymes Υ-glutamyl transferase (GGT) and alanine aminotransferase (ALT) correlate with liver fat content, and have been shown to predict impaired glucose metabolism and type 2 diabetes mellitus incidence [5]. On the other hand, secondary to insulin resistance, prolonged compensatory hyperinsulinemia may lead to the development of NAFLD [6]. From this point of view, NAFLD may be a consequence rather than a cause of age related insulin resistance.

Offspring of long-lived siblings exhibit an exceptional healthy glucose metabolism in middle age, including preservation of insulin sensitivity and increased glucose tolerance [7], [8]. We have previously shown that these subjects had a higher insulin-mediated glucose disposal rate (peripheral insulin sensitivity), while the capacity of insulin to suppress endogenous glucose production (hepatic insulin sensitivity) was not different as compared to controls [8]. In line with enhanced peripheral glucose disposal, we have previously shown that lipid accumulation within muscle cells was lower in offspring of nonagenarian siblings as compared to controls [8], [9]. Likewise, given the correlation between insulin resistance and liver steatosis [10], it can be questioned whether there is an association between the extent of liver steatosis and the healthy metabolic profile observed in familial longevity. One previous study found that nonfasting serum triglyceride levels were lower in offspring of long-lived siblings as compared to controls, albeit only in women [11]. Accordingly, while serum triglyceride levels correlate with liver fat content [12], this may suggest that the extent of liver steatosis is lower in offspring of long-lived siblings. However, triglyceride levels were determined in nonfasting samples, so results might have potentially been confounded by differences in food intake between groups. Moreover, it was previously shown that familial longevity is marked by enhanced peripheral but not hepatic insulin sensitivity [8]. Based on these considerations, we hypothesized that the extent of liver steatosis would be similar between offspring of long-lived siblings and control subjects. To test our hypothesis, we evaluated liver biochemical tests (aspartate aminotransferase [AST], ALT, and GGT) and computed tomography markers of liver steatosis in the non-diabetic offspring of long-lived siblings and age-matched controls.

## Methods

### Study Subjects

The Medical Ethical Committee of the Leiden University Medical Center approved the study, and written informed consent was obtained from all subjects according to the Declaration of Helsinki.

Subjects were included from the Leiden Longevity Study, which has been described in more detail elsewhere [13]. In short, 421 Dutch Caucasian families were enrolled in the study between 2002 and 2006 based on the following inclusion criteria: (1) there were at least two living siblings per family, who fulfilled the age criteria and were willing to participate, (2) men had to be aged ≥89 years and women had to be aged ≥91 years and (3) the sib pairs had to have the same parents. In 2002, only 0.5% of Dutch men were aged 89 and older, and only 0.5% of Dutch women aged 91 and older. Accordingly, siblings who meet these age criteria are even rarer and are estimated to represent far less than 0.1% of the population in the Netherlands [14]. Offspring of these long-lived siblings were included as they were shown to have a 35% lower mortality rate compared to the general population. Their partners, who share the same socio-economic and geographical background, were enrolled as age-matched control group [13]. Accordingly, there were no selection criteria on health or demographic characteristics.

In total, 2415 subjects comprising1671 offspring and 744 partners are included in the Leiden Longevity Study. For the current study, additional information was collected, including self-reported information on height, weight, alcohol intake and smoking habits. Information on past medical history was obtained from the participants’ treating physicians. Subjects with diabetes (65 offspring and 53 partners) were excluded. Subjects were regarded as having diabetes if they had nonfasting glucose levels >11.0 mmol/L, a previous medical history of diabetes and/or used glucose lowering agents. Of the remaining 2297 subjects, we excluded subjects with plasma levels more than threefold higher than the upper reference limit for GGT (18 offspring, 14 partners) or ALT (0 offspring, 1 partner). For the remaining subjects, all plasma AST levels were within the reference range. For the remaining 2264 subjects, plasma samples were not available for 35 subjects (25 offspring, 10 partners) and serum data on GGT or ALT were not available for 46 subjects (32 offspring, 14 partners). In total 8 subjects (8 offspring, 0 partners) were excluded based on presence of chronic hepatitis (n = 4), liver steatosis (n = 3, confirmed by ultrasound), or liver metastasis (n = 1) in past medical history. Accordingly, information on medication was lacking for 238 subjects (173 offspring, 65 partners), data on alcohol intake was missing for 293 subjects (214 offspring, 79 partners), information on smoking was lacking for 12 subjects (11 offspring, 1 partner), and information on BMI was lacking for 7 subjects (3 offspring, 4 partners). Hence, in total 1625 subjects (1122 offspring and 503 partners) were selected for the current analyses.

From the cohort of 2415 subjects, a subgroup of 234 was previously recruited, from which fasting serum samples were obtained and who participated in an oral glucose tolerance test (OGTT) [7]. From this group of 234 subjects, 4 subjects were excluded because GGT levels were more than threefold higher than the upper reference limit (2 offspring, 2 partners).

From the cohort of 2415 subjects, another random subgroup of 268 subjects was recruited for computed tomography (see below).

### Biochemical Analysis and Plasma Parameters

Fasting blood samples were obtained between November 2006 and May 2008, as previously described [7]. Nonfasting blood samples were obtained between September 2002 and May 2006. All serum and plasma measurements were performed with fully automated equipment. For insulin, the Immulite 2500 from DPC (Los Angeles, CA, USA) was applied. All other measurements were implemented on an Abbott ci8200 (Roche, Almere, the Netherlands). ALT and AST were measured using the NADH (with P-5′-P) methodology and GGT by measuring the substrate L-Gamma-glutamyl-3-carboxy-4-nitroanilide methodology. Activated reagentia were based on the optimized formulation as recommended by the International Federation of Clinical Chemistry (IFCC). Reference values were 0–55 U/l for ALT, 5–34 U/l for ASAT, and sex specific reference values for GTT were 9–36 U/l women and 12–64 U/l for males. Coefficients of variation for these measurements were all below 9%.

### Alcohol Consumption

Participants reported the number of alcoholic beverages they consumed on a weekly basis in each of the following 4 categories: beer, wine, liquor, and moderately strong alcoholic beverages such as port or sherry. Non-drinkers were considered abstainers. A drink was defined as 200 mL of beer that contained 8.0 g of alcohol, 100 mL of wine that contained 10.0 g of alcohol, 50 mL of liquor that contained 14.0 g of alcohol, or 75 mL of moderately strong alcohol types that contained 10.5 g of alcohol [15]. We added the amounts of alcohol in the four groups, and calculated the total alcohol consumption per participant in grams per day.

### Medication Use

Detailed information on medication use according to the Anatomical Therapeutic Chemical (ATC) Classification System [16] was obtained from the participants’ pharmacist. Hepatotoxic medication was defined as medication for which liver damage has been reported frequently at the National Center for Drug Safety [17].

### Image Acquisition

Unenhanced Computed Tomography examinations were performed between September 2009 and December 2010 with an Aquilion ONE (Toshiba Medical Systems, Otawara, Japan) 320 multi detector-row scanner, using the following parameters: tube voltage: 120 kV, tube current: 155 mAs, rotation time: 0.5 sec. A single cross-sectional 8-mm slice of the abdomen at the T12/L1 intervertebral disc was obtained. Imaging was performed during breath hold after expiration.

### Image Analysis

Data were processed by a research fellow (M.S.) under direct supervision of an experienced radiologist (L.K., 13 years of experience).

To quantify hepatic steatosis, we measured the attenuation of the liver in Hounsfield Units (HU) by placing a region of interest (ROI) in the right peripheral liver lobe. The ROI was made as large as possible (at least 1 cm^2^), avoiding the hepatic vessels or any focal lesions. Accordingly, as internal control, HU measures were performed in the spleen. Lower attenuation values correspond to lower tissue density, which indicates a greater fat content. One cross-sectional slice has been shown to adequately capture the majority of variance in hepatic fat content, and for a single versus three ROI measures in the liver, the intraclass coefficient has been shown 0.99 [18]. To measure liver fat content, liver-spleen ratio (L/S) was calculated, where L is the hepatic attenuation (HU) and S is the splenic attenuation (HU).

The definition of NAFLD requires that (a) there is evidence of hepatic steatosis, either by imaging or by histology and (b) there are no causes for secondary hepatic fat accumulation [1]. In previous imaging studies, hepatic steatosis has been defined as >5.5% liver fat accumulation, as assessed by magnetic resonance proton spectroscopy (^1^H-MRS) [19], [20]. Although ^1^H-MRS is the most accurate non-invasive method to quantify liver fat, CT imaging has been shown to be fairly accurate in identifying patients with moderate-to-severe liver steatosis (>30% liver fat), using histologic analysis as the reference standard [21]. Previous CT studies have defined NAFLD as L/S ratio <1 [22]. In addition, one recent study showed that L/S ratio <0.8 provides high performance in qualitative diagnosis of higher (>30%) degrees of liver steatosis with 100% specificity and 82% sensitivity using histologic analysis as the reference standard [23]. Therefore we also included this cut-off value in our analysis. In 21 subjects (7 offspring, 14 controls), ROI measurements in the spleen were not feasible (e.g. the spleen was not shown, or subjects had a splenectomy in the past medical history). These subjects were excluded from L/S ratio analyses. Accordingly, L/S ratio analysis was performed in 247 subjects.

### Statistical Analyses

Continuous variables were tested for normality and, if appropriate, logarithmically transformed and used in all calculations (LnALT, LnAST, LnGGT, LnInsulin, and LnTriglyceride). For transformed variables, data are presented as geometric means with 95% confidence intervals.

Differences in subject demographics between offspring and control subjects were calculated using student’s t-test and Pearson chi-square test. Differences in markers of lipid and glucose metabolism were assessed with the use of a linear mixed model, adjusting for age, gender, BMI, and correlation of sibling relationships. To assess the association between tertiles of liver enzymes and serum levels of glucose, linear regression analysis was performed, correcting for relation to descent (offspring of long-lived siblings or age-matched control subject), age, gender, smoking, alcohol use in g/day, and number of hepatotoxic medications. Differences in liver biochemical tests in offspring of long-lived siblings and control subjects were assessed with linear mixed model analysis, using different models. Model 1 was adjusted for age, gender, and correlation of sibling relationship. Model 2 included model 1 and was adjusted additionally for smoking, BMI, alcohol use in g/day, and number of hepatotoxic medications. Analyses were repeated after excluding all subjects with lipid-modifying agents.

Differences in CT markers of liver steatosis and NAFLD prevalence between offspring of long-lived siblings and control subjects were assessed with linear mixed models and logistic regression analysis, using the same models as in liver biochemical test analyses. For statistical analyses, Statistical Package for the Social Sciences (SPSS) software for windows (version 20.0) was used.

## Results

Subject characteristics are shown in table 1. In total, 1122 offspring of long-lived siblings and 503 controls were included for the nonfasting analyses. The offspring of long-lived siblings were slightly older than their partners (mean age 59.3 years and 58.6 years, respectively, p = 0.050). Body mass index was similar between the two groups (p = 0.25).

**Table 1:** Subject demographics.

	Nonfasting group (n = 1625)			Fasting group (n = 230)		
	Offspring	Controls	p-value	Offspring	Controls	p-value
Participants, n	1122	503		120	110	
Male gender, n (%)^‡^	521 (46%)	215 (43%)	0.17	58 (48%)	53 (44%)	0.98
Age in years (mean, SE)^†^	59.3 (0.2)	58.6 (0.3)	0.05	63.8 (0.6)	62.8 (0.7)	0.26
Alcohol consumption in g/day (mean, SE)^†^	11.7 (0.4)	11.8 (0.5)	0.83	13.8 (1.2)	13.6 (1.3)	0.91
Body mass index in kg/m^2,^ mean (SE)^†^	25.2 (0.1)	25.4 (0.2)	0.25	26.4 (0.4)	26.5 (0.4)	0.89
Hypertension yes/no, n (%)^‡^	214 (21%)	113 (25%)	0.11	26 (22%)	27 (25%)	0.47
Current smoking yes/no, n (%)^‡^	151 (13%)	74 (15%)	0.5	11 (9%)	11 (1%)	0.86
History of disease^‡^						
COPD n (%)	26 (3%)	12 (3%)	0.95	5 (4%)	6 (5%)	0.61
Stroke, n (%)	21 (2%)	13 (3%)	0.19	3 (3%)	0 (0%)	0.10
Myocard infarct, n (%)	20 (2%)	13 (3%)	0.24	1 (1%)	1 (1%)	0.94
Malignancy, n (%)	87 (6%)	34 (7%)	0.48	8 (7%)	9 (8%)	0.61
Number of hepatotoxic medications, n (%)^‡^			0.74			0.36
0	976 (87%)	444 (88%)		84 (82%)	76 (79%)	
1	129 (11%)	53 (11%)		17 (17%)	18 (19%)	
2	17 (2%)	6 (1%)		0 (0%)	2 (2%)	
3	0 (0%)	0 (0%)		1 (1%)	0 (0%)	
Use of lipid modifying agents, n (%)^‡^	76 (7%)	37 (7%)	0.67	7 (6%)	18 (16%)	0.01
Insulin in µIU/L (mean, 95% CI)^a^	15.7 (15.0–16.5)	16.5 (15.4–17.7)	0.25	5.89 (5.31–6.54)	6.74 (6.04–7.52)	0.08
Glucose in mmol/L (mean, 95% CI)^a^	5.68 (5.62–5.75)	5.87 (5.78–5.97)	0.001	5.00 (4.91–5.09)	5.13 (5.04–5.23)	0.04

Values are means (SE, standard error or 95% CI, confidence interval) or numbers (%). P values are from student’s t-test (^†^), Pearson chi-square test (^‡^), and from linear mixed model analysis, correcting for age, gender, BMI, and correlation of sibling relationship (^a^). Models were fitted for natural log-transformed values for insulin. For transformed variables, data are presented as geometric means with 95% confidence intervals.

Age: age at serum screening, Hypertension: systolic blood pressure ≥130 mmHg and/or diastolic pressure ≥85 mmHg, or administration of antihypertensive medication, COPD: chronic obstructive pulmonary disease, Insulin: nonfasting serum insulin levels, Glucose: nonfasting serum glucose levels.

Nonfasting glucose levels were lower in the offspring group as compared to control subjects (p = 0.001), while nonfasting insulin levels were not significantly different (p = 0.25). Sex specific analysis showed that compared to controls, both female offspring and male offspring had relatively lower mean nonfasting serum glucose (5.6 mmol/L vs 5.8 mmol/L, p = 0.003 for female offspring and partners respectively, and 5.8 mmol/L vs 5.9 mmol/L, p = 0.05 for male offspring and partners respectively). Subject characteristics for the fasted group are also shown in table 1. Differences between groups were comparable to those of the nonfasting group.

**Figure 1:**
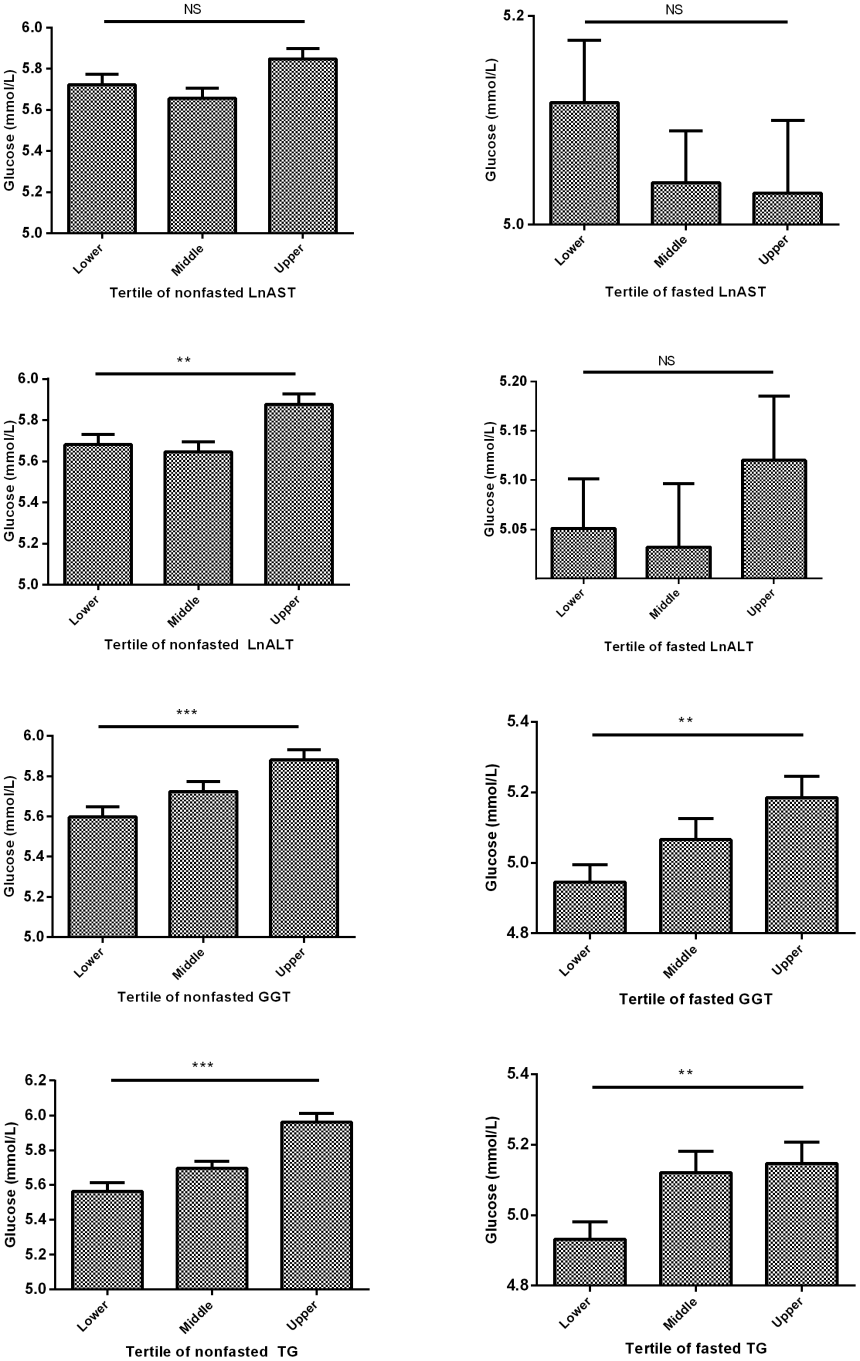
Association between liver biochemical test parameters and serum glucose. Tertiles of plasma LnALT, LnAST, LnGGT and serum LnTriglyceride (TG) (mmol/L) in association with nonfasting serum levels (mmol/L) of glucose. Asterisks (*p<0.05, **p<0.01, ***p<0.001) represents significant difference between groups using linear regression analysis, correcting for relation to sibling relationship, age, gender, smoking, alcohol use in g/day, and number of hepatotoxic medication. NS: not significant.

The association between liver biochemical tests parameters and serum TG levels with serum levels of glucose are shown in figure 1. After correcting for age, gender, smoking, use of alcohol and number of hepatotoxic medication, and correlation of sibling relationship, tertiles of nonfasting ALT (p = 0.002), GGT (p<0.001), and TG (p<0.001) were positively associated with serum levels of glucose. In the fasting group, tertiles of fasting GGT (p = 0.004) and TG (p = 0.009) were positively associated with serum levels of glucose.

Nonfasting liver biochemical tests in offspring of long-lived siblings and control subjects are shown in table 2. In both the minimally adjusted and the fully adjusted models, plasma AST and GGT were similar in offspring of long-lived siblings and control subjects. In both models, offspring of long-lived siblings had higher mean plasma ALT levels as compared to control subjects (24.3 vs 23.2 mmol/L, p = 0.03 after adjusting for age, gender, correlation of sibling relationship, smoking, BMI, and use of alcohol and hepatotoxic medications). Sex specific analysis showed that in both sexes a similar trend towards relatively higher levels of ALT in the offspring group was observed (19.9 vs 19.4 mmol/L, p = 0.27 for female offspring versus partners; 24.8 vs 21.1 mmol/L for male offspring versus partners, p = 0.12). In both the minimally adjusted and the fully adjusted models, nonfasting serumTG levels were lower in offspring of long-lived siblings and control subjects (table 2). Also after excluding all subjects with lipid-modifying agents, in both the minimally adjusted and the fully adjusted models, offspring of long-lived siblings had lower nonfasting serum triglyceride levels as compared to control subjects (offspring versus controls, 1.65 vs 1.78 mmol/L, p = 0.01 after adjusting for age, gender, correlation of sibling relationship, smoking, BMI, and use of alcohol and hepatotoxic medications). Sex specific analysis showed that after excluding all subjects with lipid-modifying agents, nonfasting triglyceride levels were lower in female offspring compared to controls (1.3 mmol/L vs 1.5 mmol/L, p = 0.001 respectively, but not in male offspring compared to controls (1.7 mmol/L vs 1.8 mmol/L, p = 0.42 respectively).

**Table 2:** Nonfasting liver enzymes and triglycerides in offspring of long-lived siblings and control subjects.

	Offspring (n = 1122)	Controls (n = 503)	p-value
ALT in mmol/L			
Model 1	22.2 (21.7–22.8)	21.2 (20.5–22.0)	0.04
Model 2	24.3 (22.8–25.9)	23.2 (21.6–24.8)	0.03
AST in mmol/L			
Model 1	26.2 (25.8–26.5)	25.9 (25.4–26.4)	0.36
Model 2	27.1 (26.1–28.1)	26.8 (25.7–27.9)	0.39
GGT in mmol/L			
Model 1	22.9 (22.2–23.6)	23.2 (22.1–24.3)	0.66
Model 2	27.9 (25.8–30.2)	27.9 (25.6–30.4)	0.96
Triglycerides in mmol/L			
Model 1	1.51 (1.47–1.56)	1.63 (1.55–1.70)	0.01
Model 2	1.55 (1.49–1.61)	1.65 (1.57–1.74)	0.02

Results are from linear mixed models, correcting for age, gender, and correlation of sibling relationship (model 1) and additionally for smoking, body mass index, alcohol use in g/day and number of hepatotoxic medications (model 2). Models were fitted for natural log-transformed values of alanine transaminase (ALT), aspartate transaminase (AST), gamma-glutamyltransferase (GGT), and LnTriglycerides. Geometric means (95% confidence interval) are reported for transformed variables.

Liver biochemical test results from the fasting group are shown in table 3. In both the minimally adjusted and the fully adjusted models, fasting ALT, AST, GGT, and triglyceride levels were similar in offspring of long-lived siblings and control subjects. Also after excluding all subjects with lipid-modifying agents, triglyceride levels were similar between the two groups (offspring versus controls, 1.51 mmol/L vs 1.52 mmol/L, p = 0.90 after adjusting for age, gender, correlation of sibling relationship, smoking, BMI, and use of alcohol and hepatotoxic medications). Sex specific analysis showed that in both sexes triglyceride levels were similar between offspring of long-lived siblings and controls.

**Table 3:** Fasting liver enzymes and triglycerides in offspring of long-lived siblings and control subjects.

	Offspring (n = 120)	Controls (n = 110)	p-value
ALT in mmol/L			
Model 1	16.3 (15.2–17.4)	15.9 (14.8–17.0)	0.64
Model 2	20.1 (15.6–25.8)	19.3 (15.0–24.9)	0.52
AST in mmol/L			
Model 1	21.2 (20.2–22.1)	20.7 (19.7–21.6)	0.45
Model 2	22.9 (19.5–27.0)	22.7 (19.2–26.8)	0.80
GGT in mmol/L			
Model 1	23.1 (21.0–25.4)	22.9 (20.7–25.3)	0.89
Model 2	35.5 (25.1–50.2)	34.8 (24.4–49.5)	0.80
Triglycerides in mmol/L			
Model 1	1.25 (1.16–1.35)	1.25 (1.16–1.36)	0.94
Model 2	1.47 (1.13–1.91)	1.47 (1.13–1.92)	0.99

Results are from linear mixed models, correcting for age, gender, and correlation of sibling relationship (model 1) and additionally for smoking, body mass index, alcohol use in g/day and number of hepatotoxic medications (model 2). Models were fitted for natural log-transformed values of alanine transaminase (ALT), aspartate transaminase (AST) and gamma-glutamyltransferase (GGT), and LnTriglycerides. Geometric means (95% confidence interval) are reported for transformed variables.

**Figure 2:**
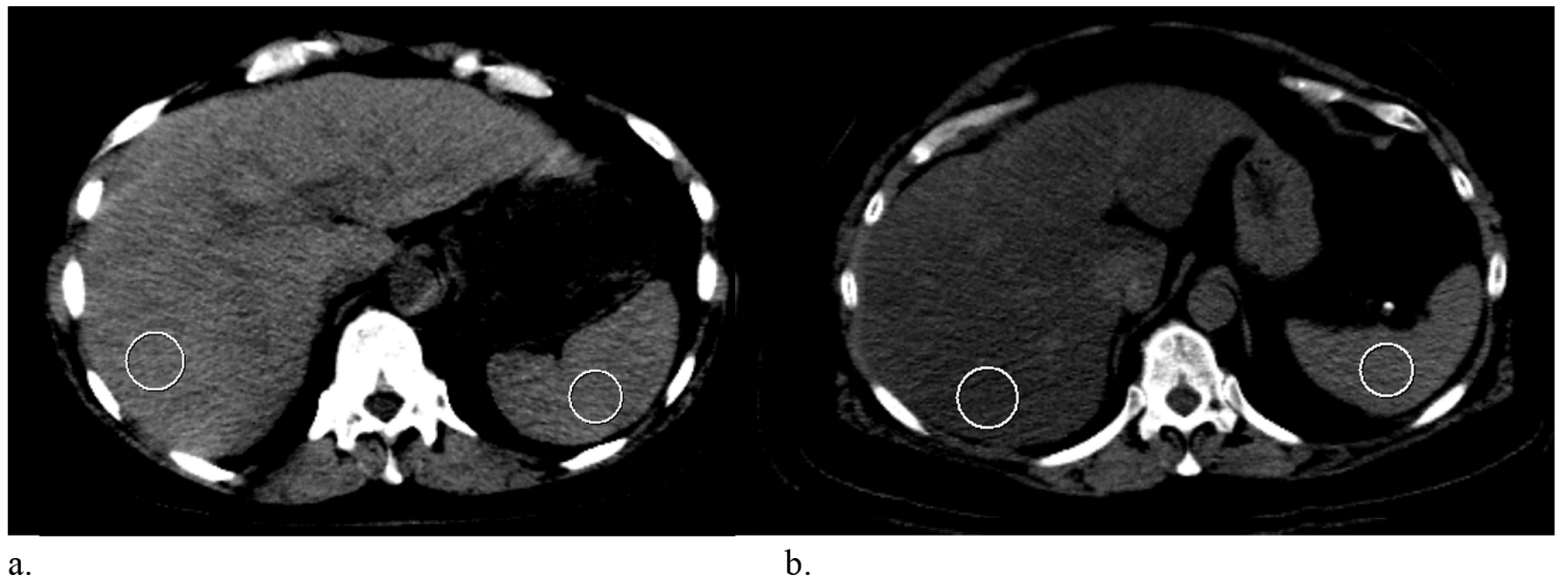
Unenhanced Computed Tomography (CT) scan of the upper abdomen, axial view. Normal liver in a 58-year-old female; liver attenuation is 62 HU, spleen is 55 HU, liver-spleen ratio is 1.1 (a). 64-year-old female with liver steatosis; the liver parenchyma (28 HU) is relatively hypodense compared to the spleen (55 HU), liver-spleen ratio is 0.5 (b). HU: hounsfield units.

Computed tomography evaluation of liver steatosis in offspring of long-lived siblings and control subjects are shown for a random subgroup in table 4 and figure 2. In the subgroup for which computed tomography data were available, nonfasting glucose levels were lower in the offspring group as compared to the control subjects, although this difference was only borderline significant (5.7 and 6.0 mmol/L for offspring of long-lived siblings and partners respectively, p = 0.06). However, nonfasting triglyceride levels (1.6 and 1.6 mmol/L for offspring and partners, respectively, p = 0.62) were not different between the two groups in the computed tomography group. In both the minimally adjusted and the fully adjusted models, absolute liver HU values and L/S ratios were not different between the two groups (p = 0.23 and p = 0.25, respectively). Also, prevalence of moderate-to-severe NAFLD was similar between offspring of long-lived siblings and control subjects (18% and 21% for offspring of long-lived siblings versus controls respectively, L/S ratio <1; 8% and 8% for offspring of long-lived siblings versus controls respectively, L/S ratio <0.8).

**Table 4:** Computed tomography markers of liver steatosis in offspring of long-lived siblings and control subjects.

	Offspring (n = 138)	Controls (n = 130)	p-value
Liver attenuation (HU)			
Model 1, mean (95% CI)	60.8 (59.2–62.3)	62.0 (60.4–63.6)	0.28
Model 2, mean (95% CI)	60.8 (58.8–62.7)	62.1 (60.1–64.0)	0.23
Liver/spleen ratio			
Model 1, mean (95% CI)	1.12 (1.09–1.16)	1.15 (1.11–1.18)	0.34
Model 2, mean (95% CI)	1.12 (1.07–1.16)	1.14 (1.1–1.18)	0.25
NAFLD (L/S ratio <1), number (%)†	29 (18%)	32 (21%)	
Model 1, OR (95% CI)	1.10 (0.54–2.26)		0.79
Model 2, OR (95% CI)	1.01 (0.51–2.25)		0.85
NAFLD (L/S ratio <0.8), number (%)†	12 (8%)	12 (8%)	
Model 1, OR (95% CI)	1.06 (0.40–2.85)		0.90
Model 2, OR (95% CI)	0.94 (0.34–2.63)		0.91

Results are from linear mixed model and logistics regression analysis, correcting for age and gender (model 1) and additionally for smoking, body mass index, alcohol use in g/day, and number of hepatotoxic medications (model 2).

† LS ratio were available in 131 offspring and 116 controls.

NAFLD: moderate-to-severe non-alcoholic fatty liver disease, L/S ratio: liver/spleen ratio, 95% CI: 95% confidence interval, HU: hounsfield units.

## Discussion

The main findings of this study are threefold. First, in a large nonfasting cohort we found that plasma ALT levels were relatively higher in the offspring of long-lived siblings as compared to controls, while nonfasting triglyceride levels were lower in offspring of long-lived siblings. In this group, plasma AST and GGT levels were similar between the two groups. Second, in our fasting subsample, all liver biochemical tests and serum triglyceride levels were similar between offspring of long-lived siblings as compared to controls. Third, computed tomography assessed liver-spleen ratios were not different between the two groups, indicating that the extent of liver fat is not different.

Consistent with the critical role for liver fat in the pathogenesis of hepatic insulin resistance and type 2 diabetes, recent studies showed that caloric restriction rapidly lowers hepatic fat content and improves hepatic insulin sensitivity in type 2 diabetes patients [10], [24]. Insulin resistance is strongly correlated with liver steatosis, and interventions that ameliorate insulin resistance result in lower insulin levels and decreased liver fat content [25]. While it has been demonstrated earlier that familial longevity is marked by better glucose tolerance and better peripheral insulin sensitivity [7], [8], our data indicate that this healthy metabolic profile is not accompanied by differences in liver fat content. In line with this notion, it was previously shown that familial longevity is marked by enhanced peripheral but not hepatic insulin sensitivity [8]. Moreover, we previously found no differences in C-reactive protein levels, a marker of systemic inflammation, between offspring of long-lived siblings and control subjects [26]. While it has been suggested that NAFLD is characterized by a systemic low-grade inflammation [27], this may also support our finding that the extent of liver fat content is similar between the two groups.

We found that nonfasting plasma ALT levels were slightly higher in offspring of long-lived siblings as compared to age-matched control subjects. This is a surprising finding as it suggests a greater liver fat content in the offspring group. One potential explanation may be that we observed a statistically but not clinically significant difference in plasma ALT levels between the two groups. An alternative explanation is that the observed difference in ALT levels is clinically significant. In line with this explanation, another recent study found that lower serum ALT levels were associated with higher all-cause mortality in old age [28]. On the other hand, in our fasting subsample, we found that fasting ALT levels were similar between the two groups. Also, we found similar levels of both fasting and nonfasting serum GGT between groups. While GGT is more strongly associated with diabetes incidence than ALT [5], it may thus be a better marker of liver fat, although ALT is considered a more liver-specific marker than GGT [5].

In the postprandial condition, triglycerides in the plasma originate mainly from intestinal derived chylomicrons and to a smaller extent from very low density lipoproteins (VLDL) [29]. Plasma triglyceride levels can increase substantially postprandially, and elevated postprandial levels, via higher peak concentrations or delayed clearance, may represent an abnormal response to an oral fat load that reflects insulin resistance [30]. Although currently under debate [31], national guidelines recommend measuring lipid levels in a fasting state. In the fasting state plasma triglyceride levels are mainly determined by triglycerides within VLDL as secreted by the liver [32]. Accordingly, results from previous studies using fasting blood samples indicate that liver fat content correlates with fasting serum triglyceride levels [12], [18], [33]. Furthermore, excessive liver fat accumulation influences the VLDL production rate [34]. In our full cohort, nonfasting serum triglyceride levels were lower in offspring of long-lived siblings as compared to controls, while nonfasting serum triglyceride levels in the CT group were similar, which is in line with the similar extent of liver fat as assessed by L/S ratios in this group. However, the CT group may thus be not fully representative for the full nonfasting group. On the other hand, in the fasting group, fasting triglyceride levels were similar, although lack of statistical power to detect a difference due to the relatively small sample size may also have been a factor. Still, based on the considerations above, we believe that liver biochemical test results from the fasting samples are in fact most indicative in assessing liver fat content.

Our data support the notion that, although healthy longevity is marked by better glucose tolerance and better peripheral insulin sensitivity, this favourable metabolic condition is not accompanied by early differences in liver fat. This strongly suggests that differences in NAFLD will only be detectable later in the pathophysiology towards type 2 diabetes pathogenesis. This notion is in line with those of a study that addressed the time sequence of the various metabolic abnormalities associated with the development of type 2 diabetes by studying their reversal after bariatric surgery [35]. Within days after surgery, liver fat levels fell and normal hepatic insulin sensitivity was restored, arguing that NAFLD is a late step in the pathophysiology towards type 2 diabetes. In contrast, muscle insulin sensitivity remained abnormal up to months after bariatric surgery, arguing that this is an earlier step and that muscle insulin resistance, caused by genetic and-or environmental factors, will facilitate the development of fatty liver at a later stage. Therefore, as previously proposed [8], it is plausible that longevity genes are involved in favourable muscle insulin sensitivity observed in familial longevity. The differences in peripheral insulin sensitivity are potentially related to the (epi)genetic differences previously observed in nutrient sensing pathways between groups [36], [37].

A strength of the current study is the large sample size which enabled us to adjust for multiple factors affecting both liver biochemical test parameters and liver attenuation at computed tomography. Also, our control group comprises partners of offspring which did not differ on any major indicators of lifestyle, including level of education, current smoking, and BMI [14].

The liver function tests we used to evaluate the extent of liver steatosis (ALT, AST, and GGT) cannot confirm a diagnosis of NAFLD or distinguish between steatosis, steatohepatitis, and cirrhosis [38]. Also, although mildly elevated ALT levels are the primary abnormality seen in NAFLD patients, liver enzymes may be normal in up to 70% of patients with NAFLD [39]. Still, in epidemiological studies, liver fat content is commonly assessed by using these liver function tests, and ALT in particular is judged to be an acceptable marker of liver fat content [40]. Although magnetic resonance proton spectroscopy is the most accurate non-invasive method to quantitate liver fat, CT imaging provides visualization of the whole liver, by which liver steatosis can be detected with high reproducibility in clinically asymptomatic individuals in the community [41]. For this purpose, CT is used to quantify liver steatosis in epidemiological studies [33], [42], [43] and clinical trials [22]. The time span between blood sampling and image acquisition is relatively large which is a potential limitation. On the other hand, we used liver biochemical test parameters and CT liver attenuation values as separate and independent (e.g. assessed at different times, with different group compositions) markers of liver steatosis in our analysis, which actually may be considered a strength of this study.

In our fasting sample we found no differences between groups in the extent of liver steatosis, as assessed with liver biochemical tests and serum triglyceride levels. These results are in line with our CT findings that liver-spleen ratios were similar between the two groups. We conclude that decreased liver steatosis is not an early metabolic phenotype that associates with the more favourable glucose metabolism in familial longevity.
